# Computational Screening of Single-Metal-Atom Embedded Graphene-Based Electrocatalysts Stabilized by Heteroatoms

**DOI:** 10.3389/fchem.2022.873609

**Published:** 2022-04-06

**Authors:** Ara Cho, Byoung Joon Park, Jeong Woo Han

**Affiliations:** Department of Chemical Engineering, Pohang University of Science and Technology (POSTECH), Pohang, South Korea

**Keywords:** heteroatom doping, metal-nitrogen-doped carbon, single-metal-atom catalysts, oxygen reduction reaction, electrocatalysts, computational screening

## Abstract

Metal-N-doped carbon is a promising replacement for non-precious-metal catalysts such as Pt for the oxygen reduction reaction (ORR) in polymer electrolyte membrane fuel cells (PEMFCs). Although these materials have relatively good catalytic activity and are cost-effective, they still have lower ORR activity than Pt, and so improving their performances is greatly required. In this study, high-throughput screening was employed based on density functional theory (DFT) calculations to search for good candidate catalysts with a transition metal atom coordinated by heteroatoms (B, N, S, O, and P) embedded in a graphene structure. In addition, coordinating a transition metal with two types of heteroatom dopants in a graphene structure was also considered. We calculated the binding energies of ORR intermediates on metal-heteroatom-based graphene structures because they are known to play a key role in ORR. Based on our results, the new group of electrocatalysts imparts excellent ORR activity for PEMFCs, and we suggest that our approach provides useful insight into exploring other promising candidate catalysts.

## Introduction

Developing high-performance oxygen reduction reaction (ORR) catalysts is a key for clean energy technologies such as fuel cells and metal-air batteries. ORR activity can be determined using the adsorption energy of oxygen intermediates as a descriptor. Although Pt invokes a high reaction rate due to its appropriate adsorption strength of oxygen intermediates (Nørskov et al., 2004), its high price and scarcity are impediments to its practical use in industry. Therefore, numerous efforts have been made to develop catalysts composed of cheap and abundant elements to replace Pt and Pt-based catalysts.

Metal single atom materials coordinated to nitrogen (M-N-Cs), especially the Fe-N-C catalyst (Lefèvre et al., 2009; Chen et al., 2019; He et al., 2020; Lu et al., 2020), have attracted research interest as the most promising alternative to Pt. Numerous studies of M-N-Cs have focused on modifying the electronic structure of the active site by controlling the coordination environment or changing the *d*-block element at the metal center. For example, Xu et al. computationally screened M-N-Cs by tuning the metal species and number of nitrogen atoms to be coordinated with the metal atom (Xu et al., 2018). They found that Fe-pyridine/pyrrole-N_4_ catalysts lying near the top of a volcano plot showed the highest ORR activity, which indicates that it would be challenging for M-N-C catalysts with only nitrogen ligands to outperform Fe-N-C catalysts. Therefore, a new strategy is required to improve ORR activity beyond the Fe-N-C catalyst.

Recently, a new approach of introducing a *p*-block element (B, N, O, P, or S) into M-N-Cs that can adjust the adsorption strength of intermediates by changing the electronic and geometric structures has been suggested. For example, Jung et al. found that hydrogen peroxide (H_2_O_2_) production rate and selectivity can be regulated by adding electron-rich or electron-poor species into Co-N-C (Jung et al., 2020). Their theoretical and experimental results demonstrated that the adsorption energy of *OOH was increased with the addition of electron-rich species such as oxygen, but decreased with the addition of electron-poor species such as protons, thereby affecting the charge amount of the metal center. Similarly, Mun et al. modulated the adsorption strength of Fe atoms in an Fe-N-C catalyst by controlling the charge density of the carbon plane via the electron withdrawing and donating properties (Mun et al., 2019). In addition, Shang et al. showed that Cu coordinated with N and S (Cu-N/S-C) formed an unsymmetrical structure with a higher ORR activity than either Cu-N-C or Fe-N-C (Shang et al., 2020). Due to their asymmetric structure, additional π-bonds between the Cu atom and the oxygen intermediates were formed, thereby enhancing the adsorption strength. Although the evidence from many studies suggests that M-N-C catalysts in which a single metal atom is coordinated with two other elements can enhance ORR activity, no one has yet examined all possible combinations.

In this work, we computationally screened all possible atomic combinations in MX_4_ and MYX_3_ structures, where M is a transition metal atom (3*d*, 4*d*, or 5*d*) and X and Y are heteroatoms (B, N, O, S, or P). First, we obtained structural stability based on the formation energy, and then the stable structures were filtered out and their limiting potentials for ORR were calculated. We found a linear scaling relationship to establish an ORR activity volcano plot from which we were able to derive several potential candidates.

## Methods

Spin-polarized density functional theory (DFT) calculations were performed by using the Vienna Ab Initio Simulation Package (VASP) (Kresse and Hafner, 1993; Kresse and Hafner, 1994; Kresse and Furthmüller, 1996a; Kresse and Furthmüller, 1996b) with projector augmented wave (PAW) pseudopotentials (Blöchl, 1994; Kresse and Joubert, 1999). Electron-exchange correlation energy values were treated with the Perdew-Burke-Enrnzerhof (PBE) functional of the generalized gradient approximation (Perdew et al., 1996). In the expansion of the plane wave, the cutoff energy was set as 400 eV. Geometry relaxation was stopped when the difference in the total force was less than 0.03 eV/Å. To avoid an artificial electrostatic field, dipole corrections were used to compute all of the energy values reported here (Neugebauer and Scheffler, 1992). In our calculations, we used semi-empirical dispersion correction of the DFT-D3(BJ) method (Grimme et al., 2011).

The MX_4_ and MYX_3_ structures were constructed based on a (5 × 5) supercell graphene with a vacuum layer of 20 Å by using Monkhorst-Pack 3 × 3 × 1 *k*-point meshes (Monkhorst and Pack, 1976). We adopted a computational hydrogen electrode (CHE) model including proton-coupled electron transfer (Nørskov et al., 2004) for the ORR free energy pathway. Based on this model, the reference electrode potential was a standard hydrogen electrode (SHE) where the free energy of protons can be related to that of 1/2 H_2_(g) under conditions of pH = 0 and 1 bar of H_2_ gas at 298 K. The Gibbs free energy of the proton-electron transfer step was given by

$$\Delta G=\Delta E+\Delta ZPE-T\Delta S+\Delta G_{U}+\Delta G_{sol}\text{,}$$

where *∆E* is the reaction energy obtained from the DFT calculations, *∆ZPE* is the change in zero-point energy, *T* is the temperature (298 K), *∆S* is the change in entropy, *U* is the applied potential to the SHE (when a potential is applied, the free energy is shifted by *∆G*_*U*_ = -*eU*, where *e* is the elementary charge of an electron), and *∆G*_*sol*_ is a solvation correction of 0.3 eV for ∗OH and ∗OOH (Xu et al., 2018).

## Results and Discussion

### Computational Screening Procedure for Metal-Heteroatom-Doped Carbon

**FIGURE 1:**
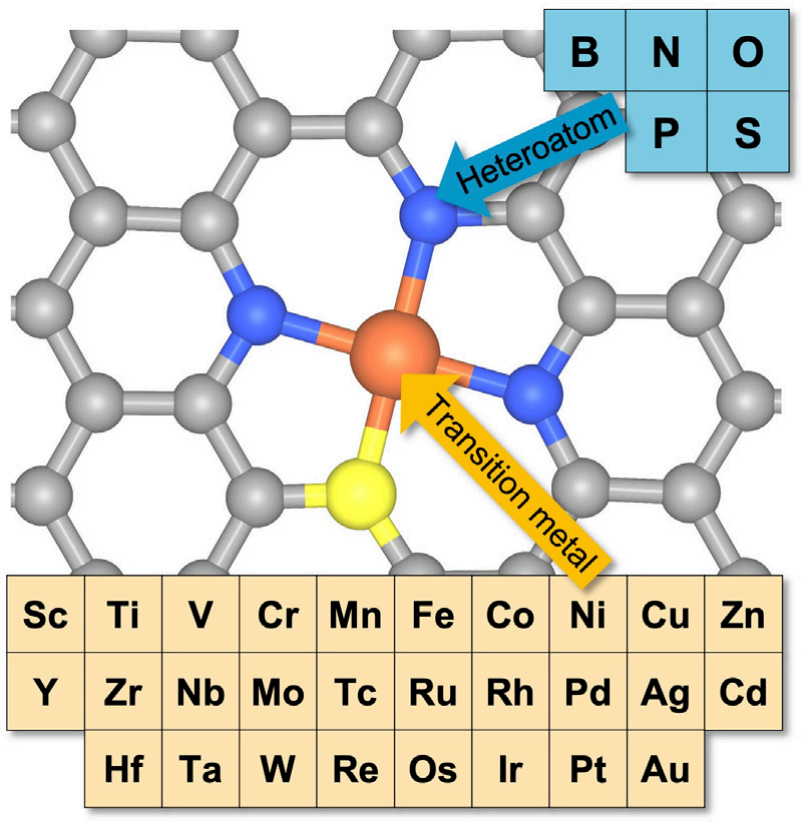
A schematic illustration of the possible combinations of metal-heteroatom-doped carbon with 28 transition metals and five heteroatoms when the coordinated heteroatoms are either the same or different at a ratio of 3:1. The gray, orange, blue, and yellow balls represent carbon, metal, host heteroatom, and dopant heteroatom, respectively.

As shown in Figure 1, we only focused on the porphyrin-like active sites where the metal center atom is coordinated with four heteroatoms in a carbon plane with two carbon vacancies due to the high computational cost. In the case of dual heteroatom doping, one of the four heteroatoms was changed, resulting in a structure with a 3:1 ratio. From the results, there were 700 possible combinations (= 28 transition metals × 5 host heteroatoms × 5 dopant heteroatoms). The variation of the *d-*orbital for transition metals ultimately increases the catalytic performance by controlling the binding strength of electrocatalytic reaction intermediates in the hydrogen evolution reaction (HER), ORR, the oxygen evolution reaction (OER), and the carbon dioxide reduction reaction (CO_2_RR) (Xu et al., 2018; Zhu et al., 2019; Park et al., 2021). Therefore, we considered 28 *d*-block transition metal elements, including 3*d* (from Sc to Zn), 4*d* (from Y to Cd), and 5*d* (from Hf to Au). Heteroatoms including B, N, O, P, and S acted as glue atoms for the transition metal in the heteroatom-doped carbon structures by changing the covalent bonding in the coordinating environment when their *p*-orbital positions were dissimilar. Moreover, local strain in the carbon lattice plane was introduced at different levels because of the different atomic sizes between the host and dopant heteroatoms.

Various forms of metal-heteroatom-doped carbon (M-YX_3_, M = transition metal, Y = dopant heteroatom, and X = host heteroatom) were considered. For example, the Co-ON_3_ structure comprises a Co atom fourfold coordinated with one O atom and three N atoms in the presence of two C vacancies within the carbon matrix. In the computational screening process, we first estimated the stability of a metal-heteroatom-doped carbon structure by considering the formation energy and single atom binding energy. Second, the free energy changes for ORR on the stable metal-heteroatom-doped carbon structures were calculated to construct a 2-D volcano plot with ΔG_OOH_ and ΔG_OH_ as descriptors to measure ORR activity.

### Structural Stability Analysis of the Metal-Heteroatom-Doped Carbon Structures

**FIGURE 2:**
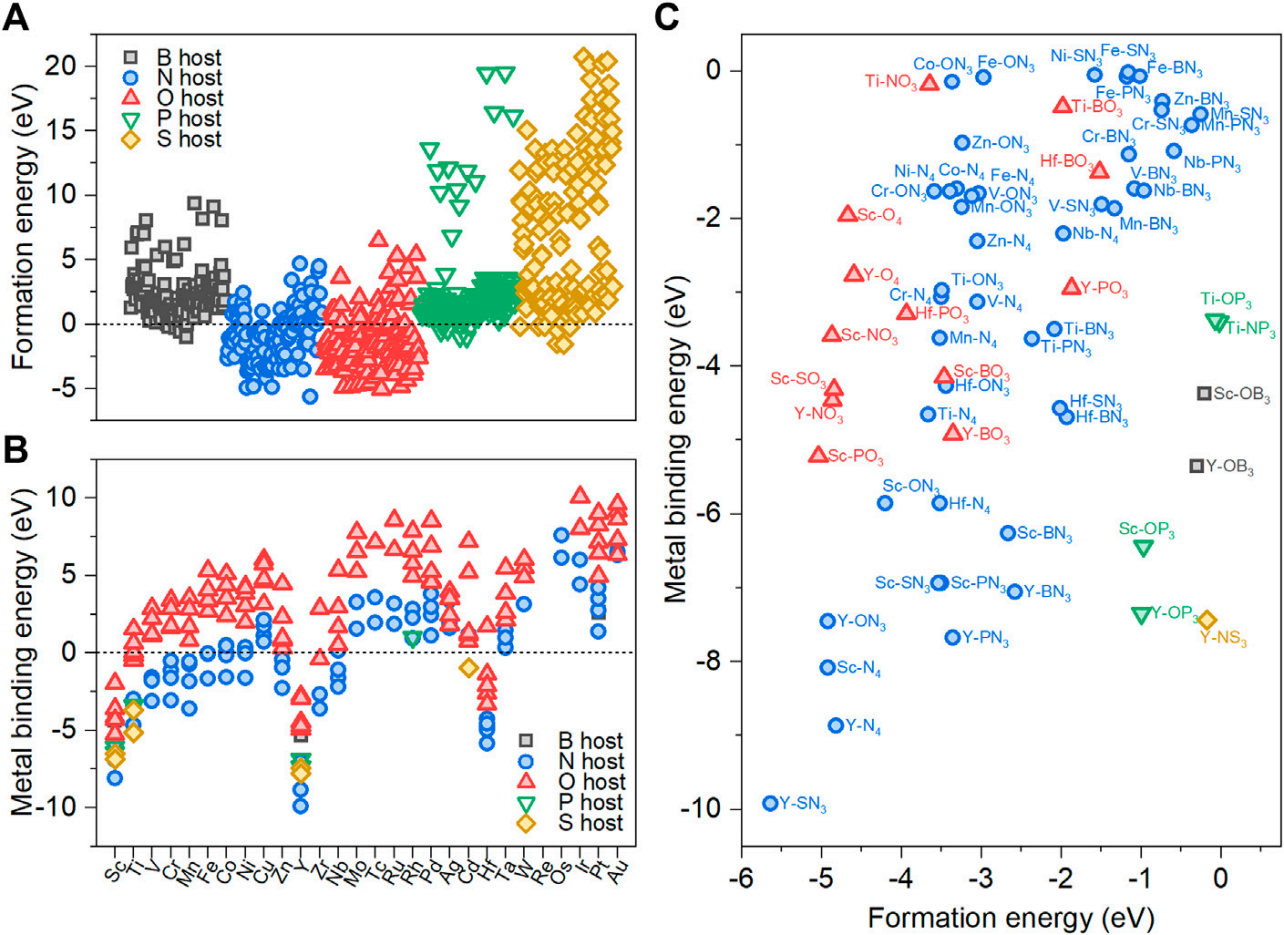
**(A)** Formation energies (*E*_form_) of 700 M-YX_3_. **(B)** Metal-binding energies (*E*_b_) of 222 M-YX_3_ with negative *E*_form_ as a function of transition metals. **(C)** Stable 66 M-YX_3_ structures with both negative *E*_form_ and negative *E*_b_.

First, we examined the stability of 700 M-YX_3_ structures by using the formation energy (*E*_form_) calculated as follows:

$$E_{\text{form}}=E_{\text{M}-{\text{YX}}_{3}}-\left(44\mu_{\text{C}}+\mu_{\text{Y}}+3\mu_{\text{X}}+E_{\text{M}}\right),$$

where $E_{\text{M}-{\text{YX}}_{3}}$ is the total energy of the optimized M-YX_3_ structure; $\mu_{\text{C}}$ , $\mu_{\text{Y}}$ , and $\mu_{\text{X}}$ are the chemical potentials of carbon, the dopant heteroatom, and the host heteroatom, respectively; $E_{\text{M}}$ is the energy per one atom of the most stable bulk metal. A negative *E*_form_ value indicates that the M-YX_3_ structure is thermodynamically stable. Figure 2A shows the results of plotting the *E*_form_ values for M-YX_3_ for each host heteroatom. The metal-heteroatom-doped carbon with N and O host heteroatoms (M-YN_3_ and M-YO_3_) had a greater number of negative *E*_form_ values compared to the other host heteroatoms. The differences in stability between the host heteroatoms are mostly attributed to the differences in their atomic sizes. Since the van der Waals atomic radii of N and O (1.55 and 1.52 Å, respectively) are smaller than C (1.70 Å) (Bondi, 1964; Mantina et al., 2009), it is possible to minimize the local strain in the carbon lattice plane, thus preventing its disruption. Because the van der Waals atomic radii of B (1.92 Å), P (1.80 Å), and S (1.80 Å) are greater than that of C, they cause local strain and disruption of the carbon lattice plane. As a result, from a total of 222 stable structures, 6 of M-YB_3_, 95 of M-YN_3_, 107 of M-YO_3_, 7 of M-YP_3_, 7 of M-YS_3_ are stable.

Next, we calculated the metal-binding energy (*E*_b_) for the 222 thermodynamically stable M-YX_3_ structures based on the formation energy. Under the applied potential for ORR, metal atoms embedded in a carbon matrix can dissolve into the electrolyte, thereby causing the matrix to lose its active sites as follows:

$$\text{M}-{\text{YX}}_{3}\to {\text{YX}}_{3}+{\text{M}}^{\text{n}+}+{n\text{e}}^{-}$$

Thus, the corresponding dissolution reaction energy at applied potential *U* was calculated by using the followed equation:

$$\text{Δ}G_{\text{M}-{\text{YX}}_{3}\to {\text{ YX}}_{3}}=G_{{\text{YX}}_{3}}+G_{{\text{M}}_{\left(\text{aq}\right)}^{\text{n}+}}+neU-G_{\text{M}-{\text{YX}}_{3}},$$

where $G_{{\text{M}}_{\left(\text{aq}\right)}^{\text{n}+}}$ is calculated from experimental standard dissolution potential *U*_*0*_ (*V*_*RHE*_) and *n* is the number of electrons transferred during the dissolution reaction. The free energy of the bulk metal (*E*_M(bulk)_) was computed as

$$G_{{\text{M}}_{\left(\text{aq}\right)}^{\text{n}+}}=E_{\text{M}\left(\text{bulk}\right)}+neU_{o}.$$

Therefore, we define the metal-binding energy using the dissolution reaction energy at operating ORR condition *U* = 0.8 V_RHE_ as follows:

$$E_{b}=-\text{Δ}G_{\text{M}-{\text{YX}}_{3}\to {\text{ YX}}_{3}}=G_{\text{M}-{\text{YX}}_{3}}-\left(G_{{\text{YX}}_{3}}+G_{{\text{M}}_{\left(\text{aq}\right)}^{\text{n}+}}+neU\right),$$

where $\text{Δ}G_{\text{M}-{\text{YX}}_{3}\to {\text{ HX}}_{3}}$ is the dissolution reaction energy. Figure 2B shows the metal-binding energy of 222 M-YX_3_ at 0.8 V_RHE_ as a function of the various transition metals. The *E*_b_ value of M-YO_3_ is more positive than that of M-YN_3_, which means that the N host heteroatoms can stabilize the metal center more readily than the O host heteroatoms. In the M-YN_3_ structures, the binding stability with the 3*d* transition metals was better than with the 4*d* and 5*d* transition metals. As a result, 66 M-YX_3_ structures were identified as stable based on the criteria of both *E*_form_ < 0 and *E*_b_ < 0 (Figure 2C).

Considering the adsorption characteristics of key reaction intermediates OOH, O, and OH during ORR is highly important for understanding the difference in electrocatalytic performance between different metal-heteroatom-doped carbon structures, and thus offers guidelines for identifying the electrocatalyst with the best ORR activity. Supplementary Table S1 summarizes all of the free energy levels of the ORR intermediates in structurally stable metal-heteroatom-doped carbon. To reduce the computational cost, we only considered the metal atom center as the active site and assumed that the associative mechanism of ORR occurs on the M-YX_3_ structure. The four-electron transfer pathway for ORR under acidic conditions, which has been well studied previously (Kulkarni et al., 2018), proceeds as follows:

$$\text{∗}+{\text{O}}_{2}+{\text{H}}^{+}+e^{-}\to {\text{OOH}}^{\text{∗}}$$

$${\text{OOH}}^{\text{∗}}+{\text{H}}^{+}+e^{-}\to {\text{O}}^{\text{∗}}+{\text{H}}_{2}\text{O}$$

$${\text{O}}^{\text{∗}}+{\text{H}}^{+}+e^{-}\to {\text{OH}}^{\text{∗}}$$

$${\text{OH}}^{\text{∗}}+{\text{H}}^{+}+e^{-}\to \text{∗}+{\text{H}}_{2}\text{O}$$

Thus, the overall four-electron pathway for ORR can be summarized as

$${\text{O}}_{2}+4{\text{H}}^{+}+4e^{-}\to 2{\text{H}}_{2}\text{O}$$

The elementary reaction step of the ORR with the maximum free energy change (*ΔG*_*max*_) value is defined as the potential-determining step (PDS). Moreover, a PDS with the positive *ΔG* value is thermodynamically unfavorable for the ORR reaction, and results in a large overpotential. The relationship between the limiting potential (*U*_*L*_) and a negative *ΔG*_*max*_ can be expressed as

$$U_{L}=-\frac{\max\left(\Delta G_{OOH}-\Delta G_{O_{2}},\Delta G_{O}-\Delta G_{OOH},\Delta G_{O}-\Delta G_{OH},-\Delta G_{OH}\right)}{e}=\frac{-\Delta G_{max}}{e}$$

**FIGURE 3:**
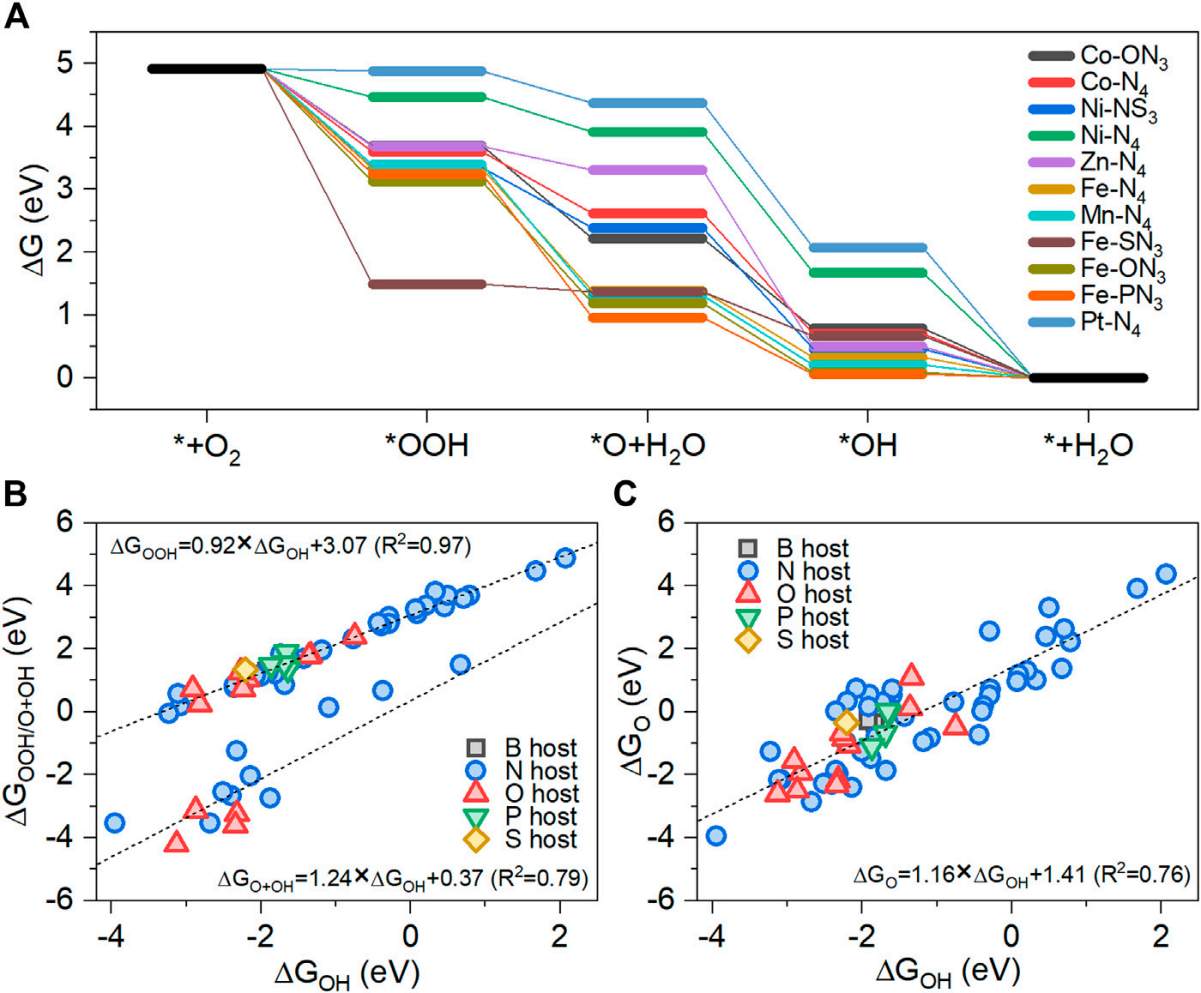
**(A)** Free energy changes during the ORR on 11 M-YX_3_ with positive limiting potential. The linear adsorption free energy scaling relationships of the ORR intermediates on the stable M-YX_3_ structures: **(B)** OOH*/O*+OH* vs. OH* and **(C)** O* vs. OH*.

The free energy diagrams for metal-heteroatom-doped carbon structures having a positive *U*_*L*_ value are shown in Figure 3A. The *U*_*L*_ means the maximum output potential at which the ORR elementary reaction steps are still exothermic (Kulkarni et al., 2018). Therefore, we selected the metal-heteroatom-doped carbon with a positive *U*_*L*_ value because the electrocatalyst with a larger *U*_*L*_ value has a higher ORR activity. The free energy changes of various metal-heteroatom-doped carbon catalysts were analyzed, for which the PDS is either OOH∗ formation or proton-electron transfer of OH∗ to form H_2_O, which is in agreement with the findings of previous research (Liang et al., 2014). Optimal adsorption strengths of the ORR intermediates are required for efficient ORR catalysis. ORR intermediates with adsorption strengths that are too weak result in insufficient O_2_ activation, and ones with adsorption strengths that are too strong result in unfavorable conditions for reducing and removing the ORR intermediates.

For M-N_4_, the *U*_*L*_ values of Co-N_4_, Fe-N_4_, Ni-N_4_, Zn-N_4_, Mn-N_4_, and Pt-N_4_ were found to be positive. The PDSs of Co-N_4_, Fe-N_4_, and Mn-N_4_ consist of the OH∗ removal step (*U*_*L*_ = 0.71, 0.33, and 0.21 V_RHE_, respectively). The OOH∗ formation step is the PDS for Ni-N_4_ and Pt-N_4_ (*U*_*L*_ = 0.45 and 0.04 V_RHE_). Thus, M-N_4_ structures with a strong OH∗ adsorption strength have the OH∗ removal step as the PDS, whereas those with a weak OH∗ adsorption strength have the OOH∗ formation step as the PDS because O_2_ activation is more difficult than in the counterparts with strong OH∗ adsorption. The PDS of the Zn-N_4_ structures was the ∗O formation step due to relatively weak O∗ adsorption strength compared to the other M-N_4_ structures. From the M-YN_3_ group, Co-ON_3_, Ni-SN_3_, Fe-ON_3_, Fe-SN_3_, and Fe-PN_3_ all showed a positive *U*_*L*_; the PDSs of Co-ON_3_, Ni-SN_3_, Fe-ON_3_, and Fe-PN_3_ were the OH∗ removal step with *U*_*L*_ = 0.79, 0.46, 0.09, and 0.07 V_RHE_, respectively, while the PDS of Fe-SN_3_ was the ∗O formation step due to relatively strong OOH∗ adsorption strength compared to the other M-N_4_ structures.

Linear scaling correlations among the ORR intermediates on the structurally stable metal-heteroatom-doped carbon structures were observed for ΔG_OOH_ or ΔG_O_ vs. ΔG_OH_. The slope and intercept for the correlation between OH and OOH were 0.92 and 3.07, respectively, with a correlation coefficient (*R* ^2^) value of 0.97, which is consistent with previous research (Calle-Vallejo et al., 2017; Kulkarni et al., 2018; Wan et al., 2019) (Figure 3B). Meanwhile, the scaling relationship between OH and O+OH was highly linear with a slope value of 1.24, an intercept value of 0.37, and an *R* ^2^ value of 0.79. Figure 3C shows the scaling relationship between O and OH. The slope and the intercept for the correlation between OH and O were 1.16 and 1.41, respectively, with an *R* ^2^ of 0.76. The O intermediates caused more oxidation of metal than OOH and OH because O species must form a double bond with the metal atom, resulting in more structural changes compared to the initial structure before the optimization. Therefore, the scaling relationship between O and OH has a larger variance than that between OOH and OH. Remarkably, for the structurally stable metal-heteroatom-doped carbon structures, the PDS is either the OOH∗ formation step or the OH∗ removal step. As shown in Supplementary Table S1, only four of the metal-heteroatom-doped carbon structures have either the O∗ formation step or the OH∗ formation step as the PDS. It is well known that the free energy changes in the OOH∗ formation step and the OH∗ removal step are determined by the relationship between ΔG_OOH_ and ΔG_OH_, and thus the *U*_*L*_ can be predicted based on this (Kulkarni et al., 2018).

### General Activity Volcano Mapping for ORR on the Metal-Heteroatom-Doped Carbon Structures

**FIGURE 4:**
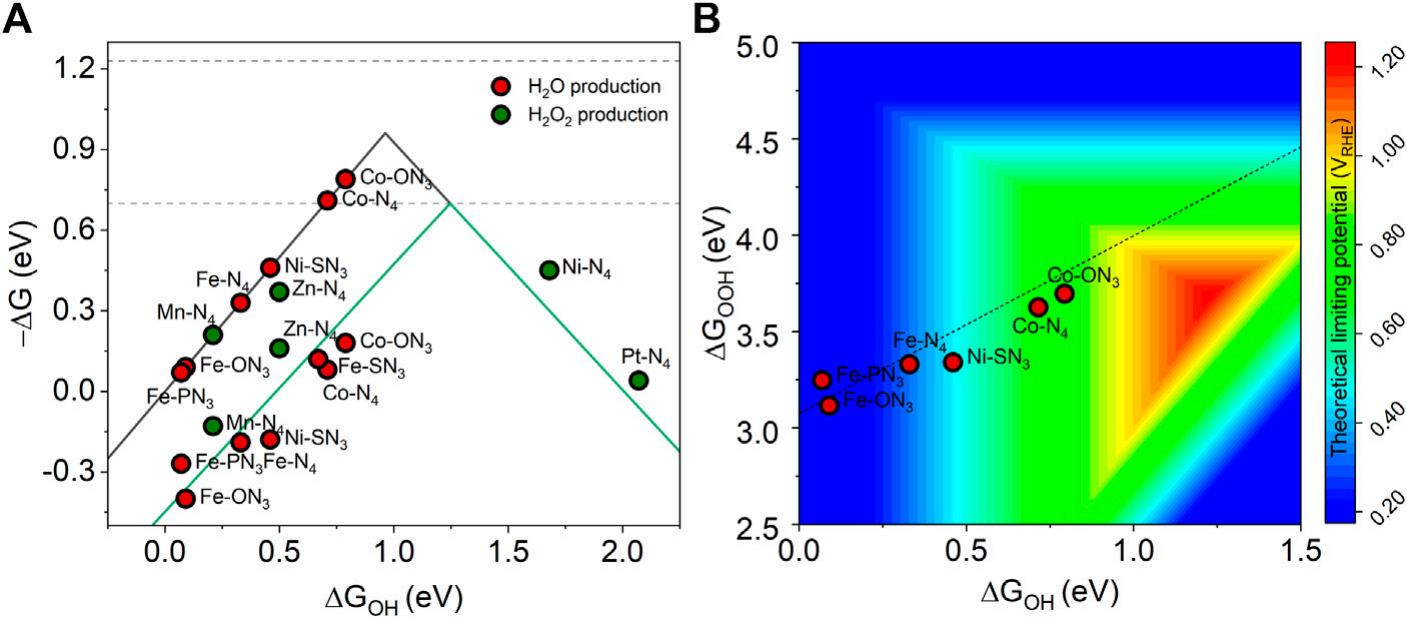
**(A)** Catalytic activity toward the H_2_O production (black solid line) and H_2_O_2_ production (green solid line), **(B)** A 2-D volcano map with ΔG_OOH_ and ΔG_OH_ as descriptors for the theoretical limiting potential of ORR on M-YX_3_ with a positive limiting potential.

We first investigated the ORR selectivity of 11 M-YX_3_ structures with a positive limiting potential. The equilibrium potential for the four and two electron pathways are shown in the dashed line at 1.23 and 0.7 eV, respectively (Figure 4A). According to the results, Mn-N_4_, Zn-N_4_, Ni-N_4_, and Pt-N_4_ had a lower overpotential of H_2_O_2_ production than H_2_O production. These results were consistent with previous theoretical findings (Jung et al., 2020). Figure 4B shows a 2-D volcano map for the theoretical limiting potential of ORR on various metal-heteroatom-doped carbon structures favoring the four-electron pathway for the H_2_O production by using ΔG_OOH_ and ΔG_OH_ as descriptors. Because *U*_*L*_ is more positive in the red area, its optimum is at ΔG_OOH_ = 3.69 eV and ΔG_OH_ = 1.23 eV. The dashed line represents the scaling relationship between ΔG_OOH_ and ΔG_OH_ (Δ_GOOH_ = 0.92 × ΔG_OH_ + 3.07). Interestingly, the O-dopant heteroatom shifted Co-N_4_ closer to the ORR optimal value (ΔG_OOH_ increased from 3.60 to 3.70 eV and ΔG_OH_ from 0.71 to 0.79 eV). When an electron-rich heteroatom species is substituted for an N atom, this positively changes the charge state of the Co atom. Thus, changing the coordinating heteroatom from -N_4_ to -ON_3_ weakens the adsorption strength of the ORR intermediates on the structure. This is because the electronegativity of O (3.44) is higher than that of N (3.04), so the metal coordinated by O is more oxidative than the one by N, resulting in the adsorption strength being weakened.

It has already been reported that Co-N-C is a good catalyst for ORR through previous DFT computational screening studies and experimental validation (Zheng et al., 2016). Using O-dopant as a heteroatom has been reported for an O-C(Al) catalyst synthesized by using isomorphic metal-organic framework MIL-53(Al, Ga) (Yang et al., 2020) or a multiwalled carbon nanotube catalyst (Jiang et al., 2019). Moreover, the role of O-dopant heteroatoms has been elucidated in Co-NG(O) catalysts (Jung et al., 2020). The Co-ON_3_ structure derived from our screening results is stable and shows the highest ORR efficiency. Based on our results and those from previous studies, we expect that it can be successfully implemented experimentally.

In this study, to simplify the procedure, we calculated the adsorption energies of the oxygen intermediates using a metal atom as the active site. However, for the materials doped with low-electronegativity heteroatoms, such as P, the oxygen may adsorb on the heteroatom rather than on the metal atom, thereby stabilizing the structure (Dipojono et al., 2019). In addition, as the applied potential increases, the oxygen intermediates can be poisoned at the active site, resulting in different adsorption configurations as well as different ORR mechanisms (Li et al., 2016). These limitations will be assessed further in the future research. Nonetheless, our work is worth noting that a large-scale screening of metal-heteroatom-doped carbon materials that include many unknown combinations was conducted and the synthesis feasibility was determined based on the set of stability criteria. Finally, we provided the ORR activity data of those stable catalysts and demonstrated an unreported promising catalyst.

## Conclusion

We have developed a computational screening process for the efficient metal-heteroatom-doped carbon ORR catalysts. M-N_4_ structures comprising 28 transition metals in conjunction with five dopant heteroatoms (B, N, O, P, and S) were constructed. Structural stability was evaluated by using the formation energy and the metal binding energy on heteroatom-doped carbon. We were able to identify 66 metal-heteroatom-doped carbon structures that were stable through rigorous stability testing. To compare the ORR activities of the 66 structurally stable metal-heteroatom-doped carbon structures, we calculated the free energy changes for ORR and selected 11 metal-heteroatom-doped carbon structures with a positive limiting potential. Finally, a 2-D volcano map constructed using ΔG_OOH_ and ΔG_OH_ as descriptors revealed that Co-ON_3_ was located nearest to the optimal point for ORR catalysis. This high-throughput screening process provides a promising path for the rational design of heterogenous electrocatalysts for energy conversion as well as scientific insight into the effect of heteroatom doping on carbon structures.

## Data Availability

The raw data supporting the conclusion of this article will be made available by the authors, without undue reservation. The coordinates of 66 structures and corresponding DFT energies used in this study are available on Catalysis-hub.org (Winther et al., 2019) under following link: https://www.catalysis-hub.org/publications/AraComputational2022.
