# Supplementary material for: Computational Screening of Single-Metal-Atom Embedded Graphene-Based Electrocatalysts Stabilized by Heteroatoms
Source: Front Chem. 2022 Apr 6;10:873609. doi: 10.3389/fchem.2022.873609 (PMC9019222; doi:10.3389/fchem.2022.873609)
Supplement: Supplementary file 1 [file Table1.pdf]

Supplementary Material

Supplementary Table 1. The free energies of ORR intermediates on the structurally stable metal-heteroatom doped carbon.

M-HX_3	ΔG_{OOH}	ΔG_O	ΔG_{OH}	U_L
Co-ON_3	3.70	2.22	0.79	0.79
Co-N_4	3.60	2.62	0.71	0.71
Ni-SN_3	3.34	2.39	0.46	0.46
Ni-N_4	4.47	3.91	1.68	0.45
Zn-N_4	3.68	3.31	0.50	0.37
Fe-N_4	3.33	1.38	0.33	0.33
Mn-N_4	3.39	1.31	0.21	0.21
Fe-SN_3	1.49	1.37	0.67	0.12
Fe-ON_3	3.12	1.19	0.09	0.09
Fe-PN_3	3.25	0.96	0.07	0.07
Pt-N_4	4.88	4.37	2.07	0.04
Mn-ON_3	2.86	0.73	-0.28	-0.28
Zn-ON_3	3.03	2.55	-0.29	-0.29
Mn-PN_3	2.80	0.53	-0.29	-0.29
Cr-ON_3	0.67	0.18	-0.37	-0.37
Cr-N_4	2.73	0.02	-0.39	-0.39
Fe-BN_3	2.82	-0.73	-0.43	-0.43
Sc-PO_3	2.40	-0.47	-0.75	-0.75
Mn-BN_3	2.32	0.30	-0.77	-0.77
Cr-BN_3	0.14	-0.83	-1.09	-1.09
Cr-SN_3	1.96	-0.95	-1.18	-1.18
Sc-SO_3	1.79	1.10	-1.34	-1.34
Y-PO_3	1.77	0.12	-1.36	-1.36
Mn-SN_3	1.68	-0.16	-1.43	-1.43
Zn-BN_3	-0.86	-1.54	-0.08	-1.46

Sc-PN_3	1.56	0.52	-1.60	-1.60
Y-PN_3	1.63	0.71	-1.60	-1.60
Sc-OP_3	1.37	-0.04	-1.64	-1.64
Y-OP_3	1.89	0.02	-1.66	-1.66
Sc-BN_3	1.48	0.23	-1.67	-1.67
V-N_4	0.86	-1.88	-1.68	-1.68
Ti-NP_3	1.54	-0.68	-1.69	-1.69
Y-BN_3	1.83	0.30	-1.73	-1.73
Ti-BN_3	1.20	-0.77	-1.81	-1.81
Sc-OB_3	1.27	-0.36	-1.83	-1.83
Ti-OP_3	1.49	-1.09	-1.87	-1.87
V-BN_3	-2.75	-1.48	-1.88	-1.88
Y-SN_3	1.33	0.54	-1.90	-1.90
Sc-SN_3	1.25	0.16	-1.91	-1.91
Y-OB_3	1.28	-0.29	-1.92	-1.92
Ti-PN_3	1.13	-1.26	-2.00	-2.00
Y-N_4	1.16	0.74	-2.07	-2.07
V-ON_3	-2.03	-2.39	-2.14	-2.14
Hf-PO_3	1.04	-1.05	-2.18	-2.18
Sc-N_4	0.96	0.33	-2.20	-2.20
Y-NS_3	1.34	-0.34	-2.21	-2.21
Hf-BN_3	0.94	-0.94	-2.22	-2.22
Sc-BO_3	0.74	-0.84	-2.24	-2.24
Y-BO_3	1.27	-0.66	-2.27	-2.27
Ti-N_4	-1.24	-1.96	-2.32	-2.32
Ti-BO_3	-3.24	-2.17	-2.32	-2.32
Ti-NO_3	-3.60	-2.32	-2.34	-2.34
Nb-BN_3	0.78	-1.88	-2.35	-2.35
Y-ON_3	0.84	0.03	-2.35	-2.35
Nb-PN_3	-2.67	-2.30	-2.40	-2.40
Ti-ON_3	-2.55	-2.27	-2.51	-2.51

Nb-N_4	-3.54	-2.86	-2.68	-2.68
Sc-NO_3	0.25	-1.93	-2.82	-2.82
Sc-O_4	-3.13	-2.49	-2.87	-2.87
Y-NO_3	0.72	-1.55	-2.91	-2.91
Y-O_4	-2.94	-2.25	-3.05	-3.05
Hf-SN_3	0.18	-2.17	-3.08	-3.08
Hf-N_4	0.57	-2.15	-3.11	-3.11
Hf-BO_3	-4.21	-2.60	-3.13	-3.13
Sc-ON_3	-0.05	-1.26	-3.23	-3.23
V-SN_3	-3.53	-3.94	-3.95	-3.95
